# Application of next-generation sequencing for rapid marker development in molecular plant breeding: a case study on anthracnose disease resistance in *Lupinus angustifolius* L.

**DOI:** 10.1186/1471-2164-13-318

**Published:** 2012-07-17

**Authors:** Huaan Yang, Ye Tao, Zequn Zheng, Chengdao Li, Mark W Sweetingham, John G Howieson

**Affiliations:** 1 Department of Agriculture and Food Western Australia, 3 Baron-Hay Court, South Perth, 6151, Australia; 2 Beijing Genome Institute – Shenzhen, Beishan Industrial Zone, Yantian District, Shenzhen, 518083, China; 3 Crop and Plant Research Institute, Murdoch University, Murdoch, 6150, Australia

## Abstract

**Background:**

In the last 30 years, a number of DNA fingerprinting methods such as RFLP, RAPD, AFLP, SSR, DArT, have been extensively used in marker development for molecular plant breeding. However, it remains a daunting task to identify highly polymorphic and closely linked molecular markers for a target trait for molecular marker-assisted selection. The next-generation sequencing (NGS) technology is far more powerful than any existing generic DNA fingerprinting methods in generating DNA markers. In this study, we employed a grain legume crop *Lupinus angustifolius* (lupin) as a test case, and examined the utility of an NGS-based method of RAD (restriction-site associated DNA) sequencing as DNA fingerprinting for rapid, cost-effective marker development tagging a disease resistance gene for molecular breeding.

**Results:**

Twenty informative plants from a cross of RxS (disease resistant x susceptible) in lupin were subjected to RAD single-end sequencing by multiplex identifiers. The entire RAD sequencing products were resolved in two lanes of the 16-lanes per run sequencing platform Solexa HiSeq2000. A total of 185 million raw reads, approximately 17 Gb of sequencing data, were collected. Sequence comparison among the 20 test plants discovered 8207 SNP markers. Filtration of DNA sequencing data with marker identification parameters resulted in the discovery of 38 molecular markers linked to the disease resistance gene *Lanr1*. Five randomly selected markers were converted into cost-effective, simple PCR-based markers. Linkage analysis using marker genotyping data and disease resistance phenotyping data on a F_8_ population consisting of 186 individual plants confirmed that all these five markers were linked to the R gene. Two of these newly developed sequence-specific PCR markers, AnSeq3 and AnSeq4, flanked the target R gene at a genetic distance of 0.9 centiMorgan (cM), and are now replacing the markers previously developed by a traditional DNA fingerprinting method for marker-assisted selection in the Australian national lupin breeding program.

**Conclusions:**

We demonstrated that more than 30 molecular markers linked to a target gene of agronomic trait of interest can be identified from a small portion (1/8) of one sequencing run on HiSeq2000 by applying NGS based RAD sequencing in marker development. The markers developed by the strategy described in this study are all co-dominant SNP markers, which can readily be converted into high throughput multiplex format or low-cost, simple PCR-based markers desirable for large scale marker implementation in plant breeding programs. The high density and closely linked molecular markers associated with a target trait help to overcome a major bottleneck for implementation of molecular markers on a wide range of germplasm in breeding programs. We conclude that application of NGS based RAD sequencing as DNA fingerprinting is a very rapid and cost-effective strategy for marker development in molecular plant breeding. The strategy does not require any prior genome knowledge or molecular information for the species under investigation, and it is applicable to other plant species.

## Background

Plant breeding is a mission of continuously discovering and pyramiding desirable genes of agronomic or end-use interest into breeding lines to produce superior cultivars. Molecular markers linked to genes of interest can be developed and applied for marker-assisted selection (MAS) to increase the efficiency of genetic improvement [1-3]. Marker development for MAS in plant breeding usually requires that a cross be made between two parental plants which differ in genes or traits of interest to produce a segregating progeny population. The genomes of these segregating plants are then fingerprinted to identify markers linked to the genes of interest. In the last three decades, a number of generic DNA fingerprinting methods, such as restriction fragment length polymorphism (RFLP) [4], random amplified polymorphic DNA (RAPD) [5,6], simple sequence repeat (SSR) [7], Diversity Arrays Technology (DArT) [8], amplified fragment length polymorphism (AFLP) [9,10] and microsatellite-anchored fragment length polymorphism (MFLP) [11-14], have been used in marker development for molecular plant breeding. These methods are effective, but are labour-intensive and time-consuming. At present, the development of markers tightly linked to genes of interest still remains a difficult task.

To expedite marker development, Michelmore *et al.*[15] described the “bulked segregant analysis” (BSA) method, in which a small number of informative segregating individual plants (usually 20) are bulked to form two pools differing only for the selection region before conducting DNA fingerprinting for identification of candidate markers linked to the genes of interest. The identified candidate markers are then tested on a large number of segregating individual plants to confirm the genetic linkage between the markers and the target genes before the markers are implemented in MAS. BSA has been widely used in marker development for molecular plant breeding [16,17]. In our experience in marker development using the DNA fingerprinting method MFLP, which is a method based on the combination of the AFLP concept with microsatellite motifs [18], we adapted the BSA principle of employing a small number of informative progeny plants, but we kept each individual plant separate in DNA fingerprinting. This approach effectively eliminated the problem of detecting “false positive” candidate markers (DNA bands appearing as candidate markers in the bulk, but proven as otherwise when tested on individual plants separately) [12,13]. Using this protocol, we have developed a number of molecular markers linked to various genes of interest applicable to plant breeding [11-14,19-24].

The next-generation sequencing (NGS) technology provides a powerful tool for detecting large numbers of DNA markers within a short time-frame. Several marker development methods utilising NGS platforms to sequence complexity reduced representations were reported, including reduced-representation libraries (RRLs) [25,26], complexity reduction of polymorphic sequences (CRoPS) [27], restriction-site associated DNA sequencing (RAD-seq) [28], sequence based polymorphic marker technology (SBP) [29], low coverage multiplexed shotgun genotyping (MSG) [30], and genotyping by sequencing (GBS) [31]. “Restriction-site associated DNA (RAD)” was originally described by Miller *et al.*[32] based on microarray platform. Baird *et al.*[33] adapted the RAD on the massively-parallel NGS platform to efficiently detect DNA polymorphisms without the requirement of any prior molecular knowledge for the species under investigation. RAD sequencing produces two types of DNA markers: one type of markers is from DNA variations within the restriction sites which are dominant markers; the other is from sequence variation adjacent to the restriction sites which are co-dominant markers [28]. RAD markers have been employed in genetic mapping on fungi [34], fish [33], insects [35], and more recently on plants [28,36,37].

Narrow-leafed lupin (*Lupinus angustifolius* L.) is a grain legume crop cultivated in Australia, Europe, America and Africa. Anthracnose caused by the fungal pathogen *Colletotrichum lupini* is the most devastating disease of lupin [38]. In Australia, a single dominant gene conferring resistance to anthracnose, designated as “*Lanr1*”, is extensively applied in the national lupin breeding program to combat the disease [12]. Two molecular markers were established using traditional marker development methods, which were linked to *Lanr1* gene at the genetic distance of 3.5 and 2.3 centiMorgan (cM), respectively [12,19]. The objectives of this research were to examine the utility of RAD sequencing, applied as DNA fingerprinting, for rapid marker development for MAS in plant breeding, and to develop molecular markers more closely linked to the disease resistance gene *Lanr1* for molecular breeding in lupin.

## Results

### Generating SNP markers by RAD sequencing

**Figure 1:**
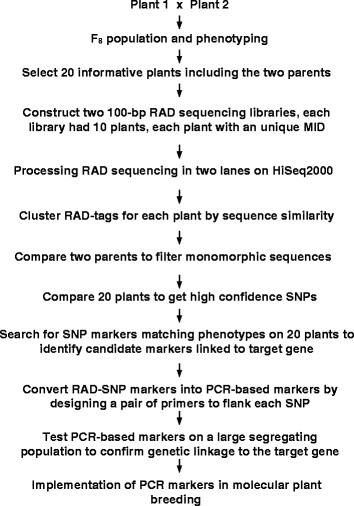
**A flow diagram illustrating the marker development procedures in this study.** The first stage was to make a cross to develop, then phenotype a genetic population. The second stage was to conduct NGS-based RAD sequencing on a small number (20) of plants representing the presence and absence of the gene of interest to generate large number of sequence reads, followed by bioinformatics analysis to identify SNP markers showing correlation between marker genotypes and plant phenotypes. The third stage was to convert SNP markers into simple PCR-based markers. Finally, the PCR-based markers were tested on a large segregating population to confirm the genetic linkage between the markers and the gene of interest before the markers were implemented in molecular plant breeding.

The marker development procedures employed in this study are illustrated in Figure 1. During the RAD sequencing stage, a total of 185 million raw reads, comprising approximately 17 Gb of sequencing data, were produced by HiSeq2000 from the two RAD-sequencing libraries constructed by the multiplex identifiers (MID) strategy from the 20 plants. After a read grouping procedure within individual plants, each plant had its tag reads for marker discovery. Tag reads from the same restriction association site in the genomes of the two parents were compared. A total of 8207 single nucleotide polymorphisms were obtained across the 20 plants in the RAD sequencing. The average coverage depth of the nucleotides of the 8207 SNP markers was 15.4X.

### Identification of candidate RAD markers linked to the *Lanr1* gene

After filtration on the 8207 SNP markers with the parameters for candidate marker identification, 38 co-dominant RAD markers were obtained (Table 1). For each of these 38 SNP markers, the nine F_8_ RIL plants with anthracnose resistance showed the polymorphic nucleotide consistent with that of the disease resistance parent Tanjil; while the nine F_8_ RIL plants susceptible to anthracnose disease exhibited the marker allele of the polymorphic nucleotide corresponding to the susceptible parent Unicrop (Table 1). These 38 RAD markers were considered as candidate markers linked to the disease resistance gene *Lanr1* based on the principles of candidate marker discovery described earlier [11-14,19-24].

**Table 1:** **Identification of 38 candidate SNP markers linked to anthracnose disease resistance gene *Lanr1* in cultivar Tanjil of *Lupinus angustifolius* L by NGS based RAD sequencing**

**Candidate markers**	**Ten plants susceptible to anthracnose disease**										**Ten plants resistant to anthracnose disease**									
	PS*	**F**_**8**_**S1**	**F**_**8**_**S2**	**F**_**8**_**S3**	**F**_**8**_**S4**	**F**_**8**_**S5**	**F**_**8**_**S6**	**F**_**8**_**S7**	**F**_**8**_**S8**	**F**_**8**_**S9**	PR*	**F**_**8**_**R1**	**F**_**8**_**R2**	**F**_**8**_**R3**	**F**_**8**_**R4**	**F**_**8**_**R5**	**F**_**8**_**R6**	**F**_**8**_**R7**	**F**_**8**_**R8**	**F**_**8**_**R9**
Candidate marker 1	a**	a	a	a	a	a	a	a	-	a	b	b	b	b	b	b	b	b	b	b
Candidate marker 2	a	a	a	a	a	a	a	a	a	a	b	b	b	b	b	b	b	b	b	b
Candidate marker 3	a	a	a	a	a	a	a	a	a	a	b	b	b	b	b	b	-	b	b	b
Candidate marker 4	a	a	a	a	a	a	a	a	a	a	b	b	b	b	b	b	b	b	b	b
Candidate marker 5	a	a	a	a	a	a	a	a	a	a	b	b	b	b	b	b	b	b	b	b
Candidate marker 6	a	a	a	a	a	a	a	a	a	a	b	b	b	b	b	b	b	b	b	b
Candidate marker 7	a	a	a	a	a	a	a	a	a	a	b	b	b	b	b	b	b	b	b	b
Candidate marker 8	a	a	a	a	a	a	a	a	a	a	b	b	b	b	b	b	b	b	b	b
Candidate marker 9	a	a	a	a	a	a	a	a	a	a	b	b	b	b	-	b	b	b	b	b
Candidate marker 10	a	a	a	a	a	a	a	a	a	a	b	b	b	b	b	b	b	b	b	b
Candidate marker 11	a	a	a	a	a	a	a	a	a	a	b	b	b	b	b	b	b	b	b	b
Candidate marker 12	a	a	a	a	a	a	a	a	a	a	b	b	b	b	b	b	b	b	b	b
Candidate marker 13	a	a	a	a	-	a	a	a	a	a	b	b	b	b	b	b	b	b	b	b
Candidate marker 14	a	a	a	a	a	a	a	a	a	a	b	b	b	b	b	b	b	b	b	b
Candidate marker 15	a	a	a	a	a	a	a	a	a	a	b	b	b	b	b	b	-	b	b	b
Candidate marker 16	a	a	a	a	a	a	a	a	a	a	b	b	b	b	b	b	b	b	b	b
Candidate marker 17	a	a	a	a	a	a	a	a	a	a	b	b	b	b	b	b	b	b	b	b
Candidate marker 18	a	a	a	a	a	a	a	a	a	a	b	b	b	b	-	b	b	b	b	b
Candidate marker 19	a	a	a	a	a	a	a	a	a	a	b	b	b	b	b	b	b	b	b	b
Candidate marker 20	a	a	a	a	a	a	a	a	a	a	b	b	b	b	b	b	b	b	b	b
Candidate marker 21	a	a	a	a	a	a	a	a	a	a	b	b	b	b	b	b	b	b	b	b
Candidate marker 22	a	a	a	a	a	a	a	a	a	a	b	b	b	b	b	b	b	b	b	b
Candidate marker 23	a	a	a	a	a	a	a	a	a	a	b	b	b	b	b	b	b	b	b	b
Candidate marker 24	a	a	a	a	a	a	a	a	a	a	b	b	b	b	b	b	b	b	b	b
Candidate marker 25	a	a	a	a	-	a	a	a	a	a	b	b	b	b	b	b	b	b	b	b
Candidate marker 26	a	a	a	-	a	a	a	a	a	a	b	b	b	b	b	b	b	b	b	b
Candidate marker 27	a	a	a	a	a	a	a	a	a	a	b	b	b	b	b	b	-	b	b	b
Candidate marker 28	a	a	a	a	a	a	a	a	a	a	b	b	b	b	b	b	b	b	b	b
Candidate marker 29	a	a	a	a	a	a	a	a	a	a	b	b	b	b	b	b	b	b	b	b
Candidate marker 30	a	a	a	a	a	a	a	-	a	a	b	b	b	b	b	b	b	b	b	b
Candidate marker 31	a	a	a	a	a	a	a	a	a	a	b	b	b	b	b	b	b	b	b	b
Candidate marker 32	a	a	a	a	a	a	a	a	a	a	b	b	b	b	b	b	b	b	b	b
Candidate marker 33	a	a	a	a	a	a	a	a	a	a	b	b	b	b	b	b	b	b	b	b
Candidate marker 34	a	a	a	a	a	a	a	a	a	a	b	b	b	b	b	b	b	b	b	b
Candidate marker 35	a	a	a	a	a	a	a	a	a	a	b	b	b	b	b	b	b	b	b	-
Candidate marker 36	a	a	a	a	a	a	a	a	a	a	b	b	b	b	b	b	b	b	b	b
Candidate marker 37	a	a	a	a	a	a	a	a	a	a	b	b	b	b	b	b	b	b	b	b
Candidate marker 38	a	a	a	a	a	a	a	a	a	a	b	b	b	b	b	b	b	b	b	b

*PS = Parental cultivar Unicrop which is susceptible to anthracnose disease; PR = parental cultivar Tanjil which is resistant to anthracnose.

**The nucleotides of the SNP markers corresponding to the disease susceptible parent Unicrop were recorded as “a”; the nucleotides of the SNP markers corresponding to the disease resistant parent Tanjil were recorded as “b”; missing data were recorded as “-”.

The DNA sequences of the 38 RAD markers were presented in Table 2. The length of the RAD reads were all 93 base pairs when the first nucleotide “G” from the *EcoRI* restriction sites (5’-G/AATTC-3’) was included. The majority of the RAD markers contained the SNP mutation sites in the middle of the RAD sequence reads (Table 2), which provided enough sequence length to design primer pairs to flank the SNP mutation sites in marker conversion.

**Table 2:** **RAD sequence reads of the 38 SNP candidate markers linked to anthracnose disease resistance gene *Lanr1* in cultivar Tanjil of *Lupinus angustifolius* discovered by RAD sequencing on NGS platform Solexa HiSeq2000**

Candidate markers*	Coverage depth**	DNA sequences (5’-3’)***
Candidate marker 1	7.0/6.4	AATTCAGATTCAAGCGTGAAATATTTATACGTAGGAGAAAATGAGAGAAGGGAGGTCTGTGAGAGAAAGAGTA[G/A]AAAGAAAAAAATAAAAAA
Candidate marker 2	5.7/6.7	AATTCAACACACCGCTCATCTCA[A/C]CTACTTTCAAAATCAACCGTCTATGGATTATCTCCACTAAGATAATATATATTATAATAAAAAATGAA
Candidate marker 3	7.1/7.5	AATTCCACAAATTGAAAAACCCGACCGCTTTTTTCATGAAATGCCAATGAAAATG[T/C]TGTTAGTACTAATACTAATTAATTGACTTCTATAAG
Candidate marker 4	11.9/13.9	AATTCCACCAGGATTGCACAACACTAATCTCAACTTGGTCTTGTTGTTTTTCATAATTGGCATCTACACCAATTG[C/G]ATATCAACACTGCTTT
Candidate marker 5	7.2/7.7	AATTCTTCTTCAGAAACAAGGAGCCAATC[G/C]GATTCCAAATGCACTGAAGGAAGCTCAAGCAGAAAGCAAAATCTAAAGAGATTAATCTGATA
Candidate marker 6	9.0/8.8	AATTCTTACAATGAACTTCTATTTTCATTTTGCACTACTAAATCATATTTGCAATATATATGTGTTATATTATGCAAACTGAATCTTT[A/G]TAT
Candidate marker 7	13.7/13.3	AATTCATCTGTACTGATTTCTTTCATATCAAATAGCAGCAGTGGCAATCCTAAAAATAGAATGACCTCT[C/T]GATGTGTGTGCATTATGTGTAT
Candidate marker 8	8.7/9.1	AATTCTTTAAACTTTTGTTCCTTTATTTGAAGTTCTCTTGCTTTTTCTAAATCAAGTAAATTAGGAGTC[A/C]TAACAAAGTTTACCTTAGAGCA
Candidate marker 9	9.1/8.8	AATTCAGAGACAGGACTCATGGCTTATTGGTAATTGGAACATT[C/T]GGAAATAGCAATGAAATCAAAGAAATTCTAGATAACCAAAAAAACACT
Candidate marker 10	15.4/15.8	AATTCTTAAAACAAATTTATACAAGTTTCTCTTTTATTCATTCATGAGAATAAGTCAAAATTGAAAATGGAAGGTAACCC[A/T]TATCAAAGCGT
Candidate marker 11	4.2/10.9	AATTCTATATCTTAGTGATTC[C/A]GTGTAACTTATACTACTAGAGGTAGGTGTGGGATGGCAATTTGACTAGTGAAATGAAGTAAAGTGACTGC
Candidate marker 12	6.8/8.5	AATTCGAACTCATATATGAATACGTGGGCATTCTTTATCAATTGAGTTAGTTGATTATT[A/T]TTTTAATTTCTTAAAAAGTTTTGAGAGCACTT
Candidate marker 13	10.9/11.4	AATTCTGATGTGAAACAACGTGAAAAGAAAGGAGAAAAATCTGTCTTCTGAACAAGAAATGGACA[G/A]ATATCAAAGCTCAGCCAGGAGCATTT
Candidate marker 14	7.42/9.24	AATTCTCTCACAAGCTAATCGACATTCATCTGTATGCTTTGC[T/A]ACAGTAGACTCTAGGACTTTCTCAAGTTCAGTCACTCTATTGCTTAGCT
Candidate marker 15	12.8/14.2	AATTCTCATAATATTTTATAGATCTCATTTAAGAGTTTAAATAGTTAG[G/C]ATAAAGTTTTTTACACATTATTATGATTAGTATTAGTATGTAT
Candidate marker 16	12.0/11.6	AATTCCCCTCAAATTAACACTGTTTCTCGTTTGGGTTCAAGAGCCCTTTGCTTATTGCTTTGAGTTTAAA[G/C]CTCCAAACTTTAAATAGAGTT
Candidate marker 17	14.9/15.6	AATTCCAGAGGATACACATGACACACTACAACATTAGTACCCG[A/G]CAATGCCTCAAAACTGCGGTCTAATATGAAAAAATCGATGTCTTTGTT
Candidate marker 18	13.3/12.5	AATTCACACTCAATCATGTTCTGCAGCTTAAACT[A/G]AAAAAACAATAGGACCTTTTGCTCTTGATAAAATTTCTGATTTAAAAAATGTACAAG
Candidate marker 19	18.9/19.2	AATTCATTCAAGGGTCTTGTCAATCAATTGA[A/C]AAAGATATATGATGAGTTGCAAGCACTTATGCTACTAAGTTCGTTGCTTGAAAACTGGGA
Candidate marker 20	12.9/13.6	AATTCTAGTTTTGTCTTGGTCCTATTGTTTGCTTGATTTTTCAATTCATTACTAAACTATT[C/T]TGACAGTTACTGCATACTATTTGCCTTAAA
Candidate marker 21	16.2/19.2	AATTCTGAAGCAAGTGTATACATTTAAGTTCTAGAAATAGAAAGGATACACTCACGG[G/A]ATGAGATAGCCAAGATAAACTATACATGGAATAT
Candidate marker 22	28.9/26.1	AATTCTAGTTTCTTTTATCTTGTTCTTTTCCCAGAAGATATTACTTGTCTTTAATTTTCTTTTGGGTGGGA[A/G]TGGGAGTGAGGGGAATTAAA
Candidate marker 23	18.7/19.8	AATTCTTTTGTTGATAACCTCAAACAAGATGGCCTAGTGTTAATCATTGGTTAGAACA[C/G]TGAAATTAATTTTTGTTTTTAAGACAACATATA
Candidate marker 24	20.2/19.8	AATTC[T/C]TAATAGGTGTAGTAGGATATATAATAAGAATACTTAAATTACTTAAAAAAGTACATAGATAGATAAATATCACTATTCGACACTCT
Candidate marker 25	14.4/16.6	AATTCTCCGTCTCTCCCCCTTCACCTTGCGGAGCAAAATCCCTCAATAGGTCCCAAGTTGACGAATCATTATCCA[C/T]CGCCGAAATCCTAATT
Candidate marker 26	7.9/7.1	AATTCTCAAGTTAAATATGATGTGGCTAACAGGGTTCACTTCTAGGTCTCGAGGTT[G/A]CTGATGCTGAAAGATCTTATCATACTGAATTGATC
Candidate marker 27	25.1/27.5	AATTCCAAATCTTTAGCTGAAAAGTCAATTAACAACTATCAACATTTTATTCAGTAGAAGTG[C/T]ATAACAGACTAGATATTGGTATTAATAAT
Candidate marker 28	21.4/20.2	AATTCATAACTACTTTGTAATTGATCAAGCTTTTCTTGCTGATCATCATTCTCTTTT[C/T]TCTCTGATTCTCTATGTTGAGTATCAAGAAATAA
Candidate marker 29	14.3/16.0	AATTCAAGCTGCTATCAACTTT[A/G]AAGCTCTCTCCCCACATGCTAACTTGATCAAATGGCTCATATATGCCCATCTTTTTCCCAATGACTAGT
Candidate marker 30	8.9/11.0	AATTCAACAACAAAAAATCATCGTAACGCCAG[T/C]AATCCTCATTGCAACAACTATAATGGCCGCAACCATAATTTAAAATACCGATTTTGTTT
Candidate marker 31	20.5/21.1	AATTCATATTCCACACAGATTTGTTCAACTGTTGAATTTGCTTACATGTCCTGAATCAAAAAGAAGAGAAAAGTTTAGACAC[G/T]ATGCGGTCA
Candidate marker 32	18.4/17.0	AATTCGAAATGGAGAGCAATGTCACTTTCATAAATGGGATAAACAAAATTTCGTTACTTAGTGCAACAGTTGGAATGCC[T/G]GTAATAACAAGC
Candidate marker 33	31.7/31.2	AATTCTGGTGCTGCGACAGAAGTGTTATGCA[A/G]AACTATTAGTCATCCTCTTAAGGTAAAACCTATTATGCTGTTACAAAAGTTCAGCTTGCC
Candidate marker 34	21.9/17.4	AATTCATTACCCTTGACA[A/C]CCTACATGAATTAGTAAAAATAAGTTTAGCCAATTCTAACATGGAACCTGTAGCATATAAAACCAATGTTCTA
Candidate marker 35	27.2/26.1	AATTCAAGCAAATTGGACCATGTGAAATCTGGATACTGTTTTGTCCCATCTTTGACAACAATAGCCTCTGG[T/G]GCTCTCAACACTTTGTCTTC
Candidate marker 36	26.5/28.8	AATTCTGCTAGTGAAGCTGG[A/G]GTTTTTCCTGCACCTGCACATTTTAGACTAGTGCCACATGAACCTGTTTGGCAATTGCCATTGCCACTTTT
Candidate marker 37	25.8/24.8	AATTCTTAGATACTTACAAGGAACAAAATAATTCGGTATATGGTATAAGACCAACACCAACTCAACATTGCACG[G/A]CTACACTAACAGTGATT
Candidate marker 38	32.2/27.6	AATTCAGTGGAATATTTCATGTTCACAAACACATTCGACCATAGCGAGAAAGTGCACCTCTC[T/A]TATTCTATTCATACCTGAATGGTTCTAAG

*Candidate marker numbers were consistent to the candidate makers listed in Table 1.

**The first number was the average coverage depth (x) of the nucleotide from the 10 plants susceptible to anthracnose disease; the second number was the average coverage depth of the nucleotide from the 10 plants resistant to anthracnose.

***The first nucleotide of each SNP in bracket for each RAD sequence read was from parental plant Unicrop; the second nucleotide of the SNP in bracket for each RAD sequence read was from parental plant Tanjil.

### Conversion of selected candidate RAD-SNP markers into PCR-based markers

**Figure 2:**
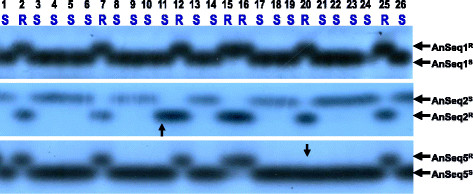
**Testing of sequence-specific PCR-based molecular markers “AnSeq1”, “AnSeq2” and “AnSeq5” on 26 F**_**8**_**recombinant inbred lines from a cross of Unicrop (susceptible to anthracnose disease) x Tanjil (resistant) of lupin (*Lupinus angustifolius L*.).** “AnSeq1^R^”, “AnSeq2^R^” and“AnSeq5^R^” indicate the marker allele bands linked to disease resistance gene *Lanr1*. “AnSeq1^S^”, “AnSeq2^S^” and “AnSeq5^S^” indicate the marker allele bands associated with disease susceptibility allele. Disease phenotypes of the RILs are presented as “S” (susceptible) or “R” (resistant). A marker band with a vertical arrow indicates that a genetic recombination occurred between the R gene and marker locus on the chromosome in that particular plant for that particular marker. All other un-marked marker bands showed the correct match between the marker genotypes and the disease resistance phenotypes on these testing plants.

Each of the five randomly selected SNP markers was successfully converted into a sequence-specific, simple PCR-based marker with a pair of sequence-specific primers flanking the SNP site (Table 3). These PCR-based SNP markers exhibited as co-dominant polymorphic bands on the SSCP gels (Figure 2). The five newly established PCR markers were designated as AnSeq1, AnSeq2, AnSeq3, AnSeq4 and AnSeq5 (Table 3).

**Table 3:** **Sequence-specific PCR markers linked to the anthracnose disease resistance *Lanr1* of *Lupinus angustifolius* developed in this study**

**Marker**	**Origin**	**Primer pair**	**Primer sequences (5’ to 3’)**
AnSeq1	Candidate marker 3*	AnSeq1F	AATTCCACAAATTGAAAAAC
		AnSeq1R	GAAGTCAATTAATTAGTATTAGTAC
AnSeq2	Candidate marker 5	AnSeq2F	CTTCTTCAGAAACAAGGAG
		AnSeq2R	CAGATTAATCTCTTTAGATTTTG
AnSeq3	Candidate marker 9	AnSeq3F	GAATTCAGAGACAGGACTC
		AnSeq3R	AGTGTTTTTTTGGTTATCTAG
AnSeq4	Candidate marker 13	AnSeq4F	GAATTCTGATGTGAAACAAC
		AnSeq4R	CTCCTGGCTGAGCTTTG
AnSeq5	Candidate marker 38	AnSeq5F	GAATTCAGTGGAATATTTCAT
		AnSeq5R	CTTAGAACCATTCAGGTATG

*Candidate marker numbers were consistent to the candidate makers listed in Table 1 and Table 2.

### Linkage confirmation between the established PCR markers and the disease resistance gene *Lanr1*

**Figure 3:**
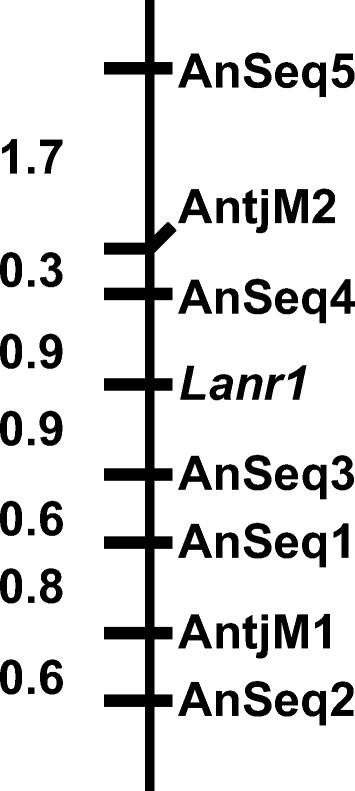
**Genetic linkage of sequence-specific PCR-based molecular markers and the disease resistance gene *Lanr1* of* Lupinus angustifolius*.** Five PCR based markers, AnSeq1, AnSeq2, AnSeq3, AnSeq4 and AnSeq5, were developed in this study. The other two markers, AntjM1 and AntjM2, which were used as controls, were established previously using traditional marker development methods [12,19]. Genetic distance in the linkage was expressed as centiMorgans. The linkage map was initially constructed using MapManager QTX [47] and finalized by RECORD program [48]. These linked markers were on linkage group “NLL-11” of the lupin genetic map reported by Nelson *et al*. [39] as evidenced by the presence of the same R gene (*Lanr*1) and the previous developed marker “AntjM2”.

Marker genotyping data were obtained for the five newly established PCR markers on 186 F_8_ RILs from the cross Unicrop x Tanjil. Linkage analysis using the marker genotyping score data and the anthracnose disease phenotyping data on the 186 F_8_ RILs showed that all the five PCR-based markers developed in this study were linked to the disease resistance gene *Lanr1* (Figure 3). The linked markers reported in this study were on linkage group “NLL-11” of the lupin genetic map reported by Nelson *et al*. [39] as evidenced by the presence of the same R gene (*Lanr*1) and the previous developed marker “AntjM2”. Three of the five markers, AnSeq1, AnSeq3 and AnSeq4, were closer to the R gene than the previously developed markers AntjM1 and AntjM2 [12,19]. Two of the newly developed markers, AnSeq3 and AnSeq4, were flanking the R gene at a genetic distance of 0.9 cM (Figure 3).

## Discussion

The marker development strategy which we applied in this study consisted of four stages. Firstly, a cross was made between two parental plants to create a segregating progeny population, followed by phenotyping of the gene of interest of the individual progeny plants. Secondly, a small number of informative plants were subjected to DNA fingerprinting by NGS based RAD sequencing to identify candidate markers linked to the target gene. Thirdly, selected candidate markers were converted into cost-effective, simple PCR-based markers. Fourthly, the converted markers were tested on a large number of individual plants of a segregating population to confirm the genetic linkage between the markers and the gene of interest before the markers were confidently implemented into a molecular breeding program. In traditional DNA fingerprinting methods such as RFLP, RAPD, AFLP and MFLP, the DNA fingerprints are visualized as DNA bands on the gels. By comparison, the “fingerprints” in RAD sequencing are presented as DNA sequence reads. SNP markers developed from RAD sequencing are suitable for high throughput multiplex implementation in molecular plant breeding on modern SNP genotyping platforms.

The most striking advantage in application of NGS based RAD sequencing as DNA fingerprinting in marker development for molecular plant breeding is the extraordinarily high efficiency. The massive power of the NGS technology for rapid and large scale marker discovery laid the foundation for the super-fast development of markers linked to the target gene *Lanr1* demonstrated in this study. In the RAD sequencing, we obtained 8207 SNP markers across the 20 test plants. This number of markers obtained from a small portion (1/8) of one sequencing run is equivalent to months of investigation with traditional DNA fingerprinting methods. The lupin genome is approximately 1540 cM [40]. The 8207 SNP markers provided an average coverage of about 5.3 markers for each cM in the genome. In theory, approximately 32 of these SNP markers would be distributed on the chromosome at either side of the *Lanr1* gene within the genetic distance of 3 cM, or 53 markers at either side of the R gene within the genetic distance of 5 cM. Therefore, it was of no surprise that 38 markers were discovered linked to the target *Lanr1* gene in this study. The large number of molecular markers associated with a target gene should provide breeders with a broad suite of options to choose the markers to suit a wide range of breeding populations to support molecular plant breeding programs [13,41,42].

A further major advantage of using NGS technology in marker development is the ease in conversion of candidate markers into cost-effective, simple PCR-based markers. In MAS, molecular markers must be cost-effectively amenable to a large number of samples [43]. In traditional DNA fingerprinting such as RAPD, AFLP and MFLP, DNA markers recovered from the gels must go through a tedious process of DNA fragment isolation, PCR amplification, cloning and sequencing to determine the DNA sequences of the marker fragments to enable the design of sequence-specific primers [6,11,18]. Sometimes marker conversion may still remain problematic even after the marker bands are sequenced, particularly for dominant markers, and for markers resulting from DNA variations from the restriction sites targeted by the restriction enzymes employed in DNA fingerprinting, in these cases further DNA sequence extension after sequencing is required [19,20]. By contrast, when NGS is used as DNA fingerprinting, the DNA sequences of candidate markers are known, and ready for primer design. With the parameters used for candidate marker identification employed in this study, all selected markers are co-dominant markers. The length of RAD sequencing reads in our study was 92 base pairs. The majority of SNP mutation sites were in the middle of the sequencing reads, which provided enough sequence length at both ends of the sequence reads to design a pair of sequence-specific primers to flank the SNP sites to convert the SNP markers into PCR-based co-dominant markers.

Anthracnose disease resistance in cultivar Tanjil of *L. angustifolius* is conditioned by a single dominant gene *Lanr1* which is highly heritable [12,19]. In this study, three out of five established sequence-specific PCR markers, AnSeq1, AnSeq3 and AnSeq4, were closer to the target gene *Lanr1* than the other two markers AntjM1 and AntjM2 previously developed with conventional DNA fingerprinting when 12 plants were used [12,19]. Two of the newly developed markers, AnSeq3 and AnSeq4, are co-dominant markers flanking the *Lanr1* gene in 0.9 cM. The accuracy to selection F_2_ plants possessing the *Lanr1* gene using either marker AnSeq3 or AnSeq4 in marker-assisted selection in lupin breeding will be approximately 99%; and the accuracy would be 99.9% if both markers are applied in MAS. Genotyping based selection using these markers is capable of distinguishing the homozygous resistant plants (*RR*) from heterozygous resistant plants (*Rr*) among the F_2_ progeny plants resulting from RxS crosses. This leads to the selection and fixation of the desirable gene at the early generation in the breeding cycle [11-14]. By comparison, plants selected based on traditional disease phenotyping would contain both the *Rr* genotype and the *RR* genotype, where further disease resistance selection in the following breeding cycle is still required due to segregation from plants with *Rr* genotype. Therefore, genotyping based marker-assisted selection is much more cost-effective than traditional phenotyping based selection. The two markers AnSeq3 and AnSeq4 are now replacing the previously developed markers AntjM1 and AntjM2 for marker-assisted selection in the Australian national lupin breeding program.

## Conclusions

We have demonstrated that the NGS-based RAD sequencing technology can be cohesively integrated into the marker development protocol for molecular plant breeding. The sequencing reads generated from the RAD sequencing have the same function and effects as the DNA fingerprints produced by traditional DNA fingerprinting methods for marker development in molecular plant breeding. The application of NGS-based technology in marker development provides several significant advantages over tradition methods. Firstly, marker development with NGS is very rapid. The entire RAD sequencing work can be completed in days. Secondly, dozens of molecular markers linked to a target gene can be discovered in one sequencing run, which is in sharp contrast to traditional DNA fingerprinting methods in which only one or a few markers can be found after working for months. The large number of linked markers not only provides the luxury for the molecular geneticist to choose the marker most closely linked to the gene, but also offers plant breeders the option to select markers applicable to a wide range of crosses in their breeding programs. Thirdly, DNA markers obtained by our marker development strategy are all co-dominant, which can readily be converted into cost-effective, simple PCR-based markers desirable for high throughput implementation on modern SNP genotyping platforms for marker-assisted selection in molecular plant breeding.

The marker development strategy applied in this study does not require any prior genome knowledge or genetic mapping information. This will potentiate its utilization across a wide range of plant species.

## Methods

### Marker development protocol

The marker development protocol used in this study was illustrated in Figure 1. The strategy contained four stages. Firstly, a cross was made to create a genetic population segregating for the gene of interest; and individual plants in the population were phenotyped. Secondly, NGS-based RAD sequencing was conducted on a small number of representative plants to identify SNP markers showing correlation between marker genotypes and plant phenotypes. Thirdly, candidate SNP markers were converted into simple PCR markers. Finally, the PCR markers were tested on a large segregating population to confirm the genetic linkage between the markers and the gene of interest; and the markers closely linked to the gene were selected and applied in molecular plant breeding (Figure 1).

### Plant materials

A single lupin plant of cultivar Tanjil (resistant to anthracnose disease) was used as the pollen donor, and was crossed with a single plant of cultivar Unicrop (susceptible to anthracnose). F_2_ seeds from a single F_1_ plant were harvested and advanced to F_8_ recombinant inbred lines (RILs) by single seed descent with no bias. The parental lines and the F_8_ population (consisting of 186 RILs) were tested against anthracnose disease in both glasshouse and field trials. Disease resistance or susceptibility in each line was assessed with the method described by Thomas *et al.*[38]. Genetic analysis for anthracnose resistance in the F_2_ and in the F_8_ populations from this cross showed that the disease resistance was controlled by a single dominant R gene, which was designated as *Lanr1*[12,19].

### Search for candidate markers linked to anthracnose resistance by RAD sequencing

The workflow for the marker development strategy in this study is illustrated in Figure 1. Selection of test plants for RAD analysis and the identification of candidate RAD markers linked to the target gene *Lanr1* followed the same principle as in marker development by MFLP [11-14,19-24]. Twenty plants were used in RAD sequencing. Ten of the plants were resistant to the disease, including the parent plant Tanjil and nine randomly selected resistant RILs. The other 10 plants were susceptible, consisting of the susceptible parent Unicrop and nine randomly selected susceptible RILs (Table 1). RAD sequencing and analyses on each of the 20 plants were treated separately.

The protocols of RAD sequencing were the same as described by Chutimanitsakun *et al.*[36], except we used the restriction enzyme *EcoRI* (recognition site: 5’-G/AATTC-3’). *EcoRI* is a more frequent cutter than the restriction enzyme *SbfI* used by Chutimanitsakun *et al.*[36], resulting in detection of more markers in RAD sequencing. Two 100 bp single-end sequencing libraries were constructed using the eight-nucleotide multiplex identifiers [33]. Each library contained 10 plants. Each plant was assigned to a unique MID barcode. The RAD products from the 20 plants together with four controls were processed in two lanes on the NGS platform HiSeq2000. Sequencing data were segregated by the individual specific MID. Reads from each plant were clustered into tag reads by sequence similarity (allowing two mismatches at most between any two reads within each tag reads cluster) and clusters with <2 or >100 reads were discarded [44].

Tag reads from the two parental plants were compared and filtered to remove monomorphic DNA sequences, leaving only the tag reads with SNP polymorphisms. The remaining sequences were then compared among all the 20 plants to select highly confident SNPs (Figure 3). All scripts used above were custom written. The algorithm of the scripts was the same as described by Catchen *et al.*[44]. The scripts used in this study are available to any researchers upon request. If a SNP marker showed the polymorphic nucleotide genotypes correlating with the disease resistance and susceptibility phenotypes on the 20 test plants, it was regarded as a candidate marker linked to the disease resistance gene based on the same principle as in candidate marker development with MFLP fingerprinting [11-14,20-24]. Any markers with more than one missing data point on the 20 plants were discarded. These selection criteria effectively eliminated all dominant markers, because dominant markers would appear on one allele, but would be absent on the other allele (sequencing reads missing either on all the resistant plants or on all susceptible plants) of the same locus [13,20,22].

### Conversion of candidate SNP markers into sequence-specific PCR markers

As a large number of candidate markers were identified linked to the *LanR1* gene in this study (Table 1), we randomly selected five candidate SNP markers for conversion into simple PCR-based markers. A pair of sequence-specific primers was designed near each end of the RAD reads for each selected candidate marker. Since all the RAD reads started from the *EcoRI* restriction sites (5’-G/AATTC-3’) (Table 2), the first nucleotide “G” from the *EcoRI* recognition sites was included in the forward primers if necessary, which was the case for markers AnSeq3, AnSeq4 and AnSeq5 (Table 3). The annealing temperature of primers was designed at approximately 54°C calculated using the nearest-neighbour model (https://www.sigmaaldrich.com). DNA fragments of converted markers were amplified in10 μl PCR consisting of 1.5 μl template DNA (approximately 100 ng), 0.5 unit of Taq polymerase (Fisher Biotec, Perth), 5 pmol each of two sequence-specific primers, 67 mM Tris–HCl (pH8.8), 2 mM MgCl_2_, 16.6 mM (NH_4_)_2_SO_4_, 0.45% Triton X-100, 4 μg gelatin, and 0.2 mM dNTPs. PCR was performed on a thermocycler (Hybaid DNA Express) with each cycle comprising 30s at 94°C, 30s at the annealing temperature (see below), and 1 min at 72°C. The annealing temperature of the first cycle was 60°C, and decreased 0.7°C in each subsequent cycle until the temperature reached 54°C. The final 25 cycles used an annealing temperature of 54°C. PCR products were resolved as single-stranded conformation polymorphisms (SSCP) [45] on 6% acrylamide gel using a Sequi-Gen GT sequencing cell (Bio-Rad). Detailed methods of running the SSCP gels were described elsewhere [46].

### Linkage confirmation between the established markers and the *Lanr1* gene

The newly established five sequence-specific PCR markers (Table 3) were tested on a segregating population consisting of 186 F_8_ RILs derived from the cross Unicrop x Tanjil. The marker genotyping score data and the anthracnose disease phenotyping data were merged and analysed by the software program MapManager QTX [47] to determine the genetic linkage between the markers and the *Lanr1* gene. The genetic distance was calculated using the Kosambi function. The linkage map was initially constructed using MapManager QTX and finalized by RECORD program [48]. The two markers, “AntjM1” and “AntjM2”, which were previously developed using traditional DNA fingerprinting methods [12,19], were included in the linkage analysis as controls.

## Abbreviations

RFLP, restriction fragment length polymorphism; RAPD, random amplified polymorphic DNA; AFLP, amplified fragment length polymorphism; SSR, simple sequence repeats; DArT, Diversity Arrays Technology; NGS, next-generation sequencing; RILs, recombinant inbred lines; RAD, Restriction-site associated DNA; MID, multiplex identifiers; MFLP, microsatellite-anchored fragment length polymorphism; BSA, bulked segregant analysis; MAS, marker-assisted selection; SNP, single nucleotide polymorphism; SSCP, single-stranded conformation polymorphisms.

## Misc

Huaan Yang and Ye Tao are authors contributed equally to this work

## Competing interests

The authors declare that they have no competing interests. 

## Authors' contributions

MWS and JGH provided supervisory roles. HY and CL designed experiments and interpreted the genetics of the sequencing data. TY and ZZ performed the RAD sequencing and bioinformatics. HY created the F_8_ RIL plant population, phenotyped the plants, prepared the DNA, did the marker tests on the RIL population, and drafted the manuscript. All authors have read and approved the final version of this manuscript.
